# Exceptional Longevity and Polygenic Risk for Cardiovascular Health

**DOI:** 10.3390/genes10030227

**Published:** 2019-03-18

**Authors:** Mary Revelas, Anbupalam Thalamuthu, Christopher Oldmeadow, Tiffany-Jane Evans, Nicola J. Armstrong, Carlos Riveros, John B. Kwok, Peter R. Schofield, Henry Brodaty, Rodney J. Scott, John R. Attia, Perminder S. Sachdev, Karen A. Mather

**Affiliations:** 1 Centre for Healthy Brain Ageing, School of Psychiatry, UNSW Medicine, University of New South Wales, Sydney, NSW 2031, Australia; maryg922@gmail.com (M.R.); a.thalamuthu@unsw.edu.au (A.T.); N.Armstrong@murdoch.edu.au (N.J.A.); h.brodaty@unsw.edu.au (H.B.); p.sachdev@unsw.edu.au (P.S.S.); 2 Neuroscience Research Australia, Randwick, NSW 2031, Australia; john.kwok@sydney.edu.au (J.B.K.); p.schofield@neura.edu.au (P.R.S.); 3 Hunter Medical Research Institute, Newcastle, NSW 2305, Australia; christopher.oldmeadow@newcastle.edu.au (C.O.); tiffany.evans@hmri.org.au (T.-J.E.); carlos.riveros@hmri.org.au (C.R.); John.Attia@newcastle.edu.au (J.R.A.); 4 Discipline of Mathematics and Statistics, Murdoch University, Perth, WA 6150, Australia; 5 Faculty of Health, University of Newcastle, Newcastle, NSW 2308, Australia; rodney.scott@newcastle.edu.au; 6 School of Medical Sciences, University of New South Wales, Sydney, NSW 2052, Australia; 7 Dementia Centre for Research Collaboration, University of New South Wales, Sydney, NSW 2052, Australia; 8 Pathology North, John Hunter Hospital, Newcastle, NSW 2305, Australia; 9 Neuropsychiatric Institute, Prince of Wales Hospital, Barker Street, Randwick, NSW 2031, Australia

**Keywords:** polygenic risk score, cardiovascular health, exceptional longevity, lipid profile

## Abstract

Studies investigating exceptionally long-lived (ELL) individuals, including genetic studies, have linked cardiovascular-related pathways, particularly lipid and cholesterol homeostasis, with longevity. This study explored the genetic profiles of ELL individuals (cases: *n* = 294, 95–106 years; controls: *n* = 1105, 55–65 years) by assessing their polygenic risk scores (PRS) based on a genome wide association study (GWAS) threshold of *p* < 5 × 10^−5^. PRS were constructed using GWAS summary data from two exceptional longevity (EL) analyses and eight cardiovascular-related risk factors (lipids) and disease (myocardial infarction, coronary artery disease, stroke) analyses. A higher genetic risk for exceptional longevity (EL) was significantly associated with longevity in our sample (odds ratio (OR) = 1.19–1.20, *p* = 0.00804 and 0.00758, respectively). Two cardiovascular health PRS were nominally significant with longevity (HDL cholesterol, triglycerides), with higher PRS associated with EL, but these relationships did not survive correction for multiple testing. In conclusion, ELL individuals did not have significantly lower polygenic risk for the majority of the investigated cardiovascular health traits. Future work in larger cohorts is required to further explore the role of cardiovascular-related genetic variants in EL.

## 1. Introduction

The human life span has significantly increased over the last century with many individuals surpassing 80 years of age in developed countries due to factors such as improved healthcare and favourable lifestyle choices [1]. Exceptional longevity, defined as exceeding the average life expectancy, is multifaceted with genetic, environmental and epigenetic factors all playing a role. Exceptionally long-lived (ELL) individuals are examples of successful ageing with a proportion demonstrating compression of morbidity [2]. Thus, ELL individuals have been described as “super controls” for studies on age-related decline and disease [3]. It is important to study these models of successful ageing, as these rare individuals may reveal novel longevity-associated pathways, which may ultimately translate into strategies to promote health in our ageing population.

There is evidence linking healthier cardiovascular risk profiles and lower incidence of cardiovascular disease with longevity [4]. Analysis of lipid metabolism in longevous families identified changes in lipid concentration, specifically a smaller total cholesterol to high-density lipoprotein-cholesterol (TC/HDL-C) ratio and lower triglycerides levels, in the offspring of nonagenarians [5]. Lipid profiling in the Leiden Longevity Study established larger low-density lipoprotein particles as major predictors of longevity [5]. Similarly, Barzilai et al. (2015) suggested that healthy ageing is promoted by a unique lipoprotein profile [6]. Levels of apolipoproteins, important lipid transporters in the circulatory system, have been observed to decline with age. However, higher apolipoprotein levels in the exceptionally long lived have been reported, suggesting a younger apolipoprotein profile that may promote longevity [7]. 

Further evidence from candidate and genome-wide association longevity studies indicates that cardiovascular pathways are involved in successful ageing. In a parental longevity genome wide association study (GWAS) examining 75,000 participants, the authors concluded that cardiovascular-related pathways are important contributors in attaining exceptional longevity [8]. In a recent meta-analysis examining longevity genetic polymorphisms, all significant genes (*APOE*, *FOXO3A*, *ACE*, *Klotho* and *IL6*) play roles in cardiovascular pathways, such as lipid metabolism, or have been previously linked to cardiovascular disease [9]. The genetic variants with the largest effect sizes in this meta-analysis were located in the *APOE* and *FOXO3A* genes. The product of the *APOE* gene transports lipids in the blood and is consequently critical in cholesterol metabolism. Any loss of cholesterol homeostasis may increase the risk of cardiovascular disease, obesity and diabetes [10]. In addition, cardiovascular diseases (heart attack and stroke) have been strongly linked to *APOE* [11]. *FOXO3A* is an evolutionary conserved transcription factor and has been consistently and independently replicated with longevity in many ethnically diverse cohorts [12]. Recently, the longevity-associated G allele of *FOXO3* rs2802292 in older Japanese and Caucasians was associated with decreased risk of coronary artery disease mortality [13]. Interestingly, both Ashkenazi Jewish and Italian centenarians genotyped for an isoleucine to valine variation at codon 405 in the cholesteryl ester transfer protein (*CETP*) gene had a higher frequency of the VV genotype, which has been associated with larger low-density lipoprotein particle sizes and lower *CETP* serum levels. The authors deduced that lipoprotein particle size is heritable and encourages healthy aging [6,14]. Similarly, homozygosity for the -641 C allele in the *APOC3* promoter (rs2542052) was 25% higher in centenarians and linked with pro-longevity lipoprotein levels and sizes [15]. Additional prior studies investigating genetic vascular factors involved in human longevity have described variants in genes involved in blood pressure regulation such as methyltetrahydrofolatereductase (*MTHFR*), paraoxonase 1 (*PON1*) and plasminogen activator inhibitor type I (*PAI-1*) [16]; however, further studies are still needed to confirm these suggested associations. 

Polygenic risk scores (PRS) for cardiovascular-related phenotypes can now be calculated due to the availability of summary data from GWAS examining a broad range of traits from lipids to coronary artery disease. This facilitates the evaluation of the contribution of polygenic risk for cardiovascular risk factors and disease to exceptional longevity and successful ageing. Thus, the purpose of this study was to explore the genetic profiles of ELL individuals aged (≥95 years) by assessing their polygenic risk for cardiovascular-related risk and disease phenotypes relative to middle-aged controls. This study tests the hypothesis that ELL individuals have lower polygenic risk for cardiovascular health-related traits and disease compared to controls, which may give them a survival advantage. 

## 2. Materials and Methods 

### 2.1. Participants 

ELL individuals were recruited from two Sydney-based Australian studies: The Sydney Centenarian Study (SCS) [17] and the Sydney Memory and Ageing Study (Sydney MAS) [18]. Both studies recruited participants using the compulsory electoral roll and Medicare lists from New South Wales, Australia. A subsample from SCS with available genetic data provided 256 long-lived cases with a European background (age range 95–106, mean age 97.5 years, 31% male). This sample was enriched by the addition of 38 individuals from Sydney MAS (≥95 years, mean age 86.7 years, 26% male). Ethics approval was granted from the relevant Human Research Ethics Committees for each study. SCS and Sydney MAS were approved by the Human Research Ethics Committees of UNSW Sydney and the South Eastern Sydney and Illawarra Area Health Service (ethics approvals HC17251 and HC14327, respectively). The Hunter Community Study (HCS) was approved by the University of Newcastle and Hunter New England Human Research Ethics Committees (HREC 03/12/10/3.26). Written informed consent was obtained from all participants or if unable to consent, proxy consent was obtained from the nearest of kin. 

Controls were obtained from the Hunter Community Study (HCS) in Newcastle, New South Wales [19]. These participants have a similar ethnic background to the long-lived cases and Newcastle is geographically close to Sydney (160 km). The HCS is a cohort of 3253 individuals (age range 55–85, mean age 66.3 years, 46% male). For the purpose of this investigation a subsample of 1105 individuals aged 55–65 (mean age 60.3 years, 47% male) were used as controls. 

Fasting blood samples from each cohort were collected for DNA and biochemistry analyses. Biological measures assessed included total cholesterol, as well as low-density lipids (LDL) and high-density lipids (HDL), and triglycerides as described in [20,21]. 

### 2.2. Genotyping 

DNA was extracted using standard methods. SCS cases were genotyped using the Illumina OmniExpress array (California, USA), whereas the HCS cases were genotyped using the Affymetrix Axiom Kaiser array (California, USA) and the Sydney MAS samples were genotyped using the Affymetrix Genome-wide Human SNP Array 6.0 according to the manufacturer’s instructions. In all three cohorts, genotyped single nucleotide polymorphisms (SNPs) were excluded if the following criteria were observed: (i) The call rate was <95%, (ii) *p*-value for Hardy-Weinberg equilibrium was <10^−6^, (iii) minor allele frequency was <0.01% and (iv) the strand ambiguous (A/T and C/G). If first- or second-degree relatives were identified, only one family member was retained for analysis. EIGENSTRAT analysis [22] allowed for the detection and removal of any ethnic outliers. After quality control (QC) checks, for SCS and Sydney MAS there were genotyping data on 640,355 and 734,550 SNPs, respectively, whilst for HCS there were data on 739,276 SNPs. As part of the QC checks, the reported sex of the participants was verified using genotyped data and samples with sex discrepancies were discarded. The quality controlled genotype data were imputed in the Michigan imputation server (https://imputationserver.sph.umich.edu) [23] using the Haplotype Reference Consortium reference panel (v3.20101123). Similar to the genotyped data, QC steps in the imputed data were implemented (minor allele frequency (MAF) > 0.05, imputation quality score >0.6, call rate >0.95, HWE *p*-value >10^−6^). The SNPs with high quality dosage scores were converted to best-guess genotypes using PLINK and were used in the calculation of the PRS. The genotypes for the *APOE* single nucleotide polymorphisms (SNPs) rs7412 and rs429358 were extracted from the imputed dosage using PLINK. Both the SNPs were imputed with high accuracy in all the three cohorts (r2 > 0.80) and the *APOE* ε2/3/4 haplotypes were inferred using these two SNPs [24]. 

Genotyping availability: Due to ethical concerns, genotyping data have not been deposited in a public repository. However, genotyping data can be requested via a formal review process to the relevant studies. 

### 2.3. Polygenic Risk Scores (PRS)

PRS were generated by using the PRSice program [25] from summary statistics obtained from previous GWAS studies. The following phenotypes were examined: Longevity, cardiovascular disease (myocardial infarction, all stroke and coronary artery disease) and cardiovascular disease-related risk factors (cholesterol, triglycerides, high- and low-density lipoproteins and essential hypertension). Details regarding each GWAS utilised, including links to the summary data are provided in the Supplementary (Tables S1 and S2). Linkage disequilibrium pruning was performed using the clumping option (r2 > 0.25 and physical distance threshold of 250 kb KB). PRS were calculated for each of the phenotypes using different *p*-value significance cut-offs for the SNPs included from each of the GWAS, ranging from *p* = 5 × 10^−8^ to 1. We only present the results for the PRS *p*-value threshold of 5 × 10^−5^ in the main text. Due to the strong associations between longevity and (i) the *APOE* locus (chromosome 19, base pair 45,393,826–45,422,606) and (ii) the *FOXO3A* locus (chromosome 6, base pair 108,881,038–109,005,977), longevity PRS were also calculated after removing these loci.

### 2.4. Statistical Analyses 

All analyses were performed using R version 3.4.3 [26]. Chi-squared tests were utilised to determine differences in proportions between groups. To achieve normality of the lipid variables, inverse normal transformations were performed. Independent sample t tests were used to compare the means between the cases and controls. 

To demonstrate that the PRS were associated with their respective phenotypes in our sample, analyses were undertaken investigating the relationships between (a) PRS for EL phenotypes (+/−*APOE*, *FOXO3A* and *APOC3* loci) and EL in our sample using logistic regression, controlling for sex and where appropriate *APOE* ε4 or *APOC3* (rs2542052) C homozygotes; (b) cardiovascular risk factor (lipids) PRS and measured lipid levels using linear regression, adjusting for age and sex and where appropriate *APOE* ε4 or *APOC3* (rs2542052) C homozygotes. Lastly, logistic regressions were used to investigate associations between cardiovascular health PRS and EL in our sample, controlling for sex. 

The variance explained by the PRS using logistic regression was calculated using the Nagelkerke method [27] as implemented in the R package rsq [28] and power calculation was performed using WebPower [29]. Correlation analyses between the different PRS were undertaken to examine genetic overlap, using the PRS calculated at the nominated GWAS threshold *p*-value cut-off of 5 × 10^−5^.

## 3. Results

### 3.1. Sample Characteristics 

The sample characteristics are described in Table 1. As expected, there were fewer *APOE* ε4 carriers in the ELL cases compared to the younger controls, 14.6% versus 30.8%, respectively (*p* = 1.49 × 10^−8^). LDL and total cholesterol levels were statistically different, with controls displaying higher lipid levels than EL cases (Table 1). The frequency of *APOC3* (rs2542052) C homozygotes did not differ significantly in the cases and the controls (*p* = 0.503564).

### 3.2. Polygenic Risk Scores (PRS)

The exceptional longevity and cardiovascular risk and disease phenotypes used for calculation of the PRS are shown in Table 2. The total number of SNPs included from each GWAS for the PRS calculations are listed for two GWAS p-thresholds (genome-wide significance *p* < 5 × 10^−8^, suggestive *p* < 5 × 10^−5^). Details for all other calculated PRS calculated (at different *p*-value cut-offs) are available in the Supplementary (Table S3). 

### 3.3. PRS Associations with Measured Phenotypes

#### 3.3.1. Longevity PRS with Exceptional Longevity 

**Figure 1:**
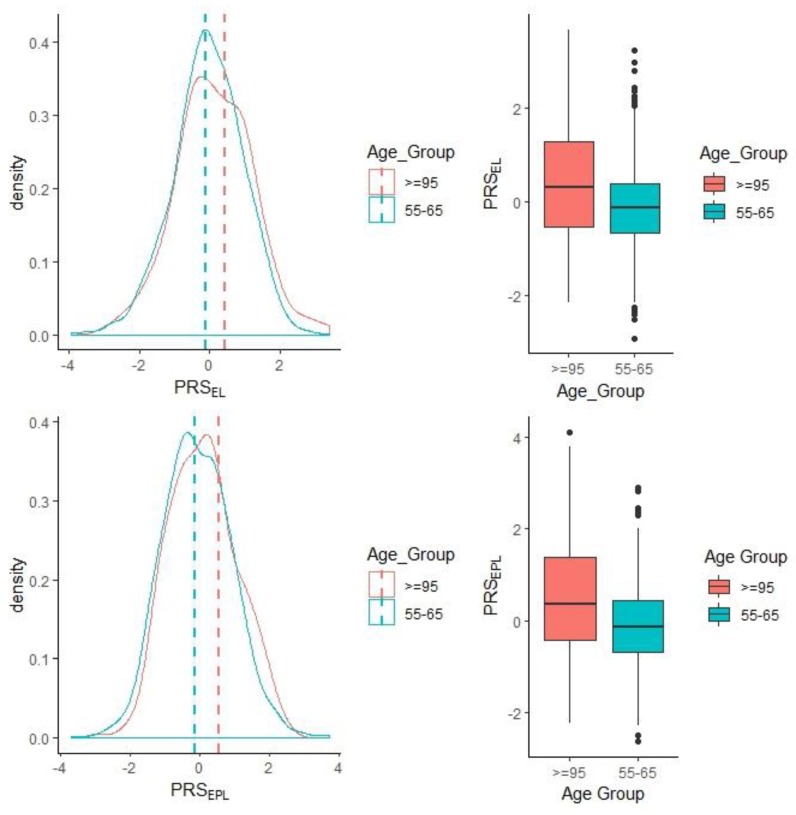
Density plots visualizing the standardised PRS distributions for EL (exceptional longevity, PRSEL) and EPL (exceptional parental longevity, PRSEPL) in the entire sample and the corresponding box plots in the long-lived cases (95+ years) versus controls (55–65 years).

As expected, higher longevity PRS from either Broer et al. [30] (EL, ≥90 cases vs. controls) or Pilling et al. [8] (exceptional parental longevity—EPL cases had a mother who lived ≥98 years and a father ≥95 years vs. controls) were significantly associated with longevity in our sample (Table 3, Figure 1). Results from both analyses suggest individuals carrying pro-longevity variants were 1.19–1.20 times more likely (per standard deviation increase in PRS) to survive to an exceptional age (≥95 years) or to have both parents that were ELL. PRS calculated at other *p*-value thresholds were all statistically significant (Table S4). For example, at a *p*-value threshold of 0.05, the EL PRS had an OR of 1.79 (*p* = 2.29 × 10^−17^), and the PRS for exceptional parental longevity had an OR of 2.01 (*p* = 6.34 × 10^−23^). Sex was also found to be significant. Figure 1 shows that within the ELL cases, there is a subsample that have low-longevity PRS (i.e., for both EL and EPL) and yet have survived to 95 years old and over. The PRS explained 0.7%–10% of the phenotype variance depending on the GWAS *p*-value threshold (Table S4).

Additional analyses excluding the *TOMM40/APOE/APOC1* locus (Table S5), the *FOXO3A* locus (Table S6) and the *APOC3* locus (Table S7) did not markedly change the results. When adjusting for ε4 carrier status or *APOC3* C homozygotes, the results did not change noticeably (Tables S8 and S9). 

#### 3.3.2. Association of Cardiovascular Risk Factor (Lipids) PRS with Measured Lipid Levels

Linear regressions were performed to ascertain whether the PRS for the lipid cardiovascular risk factors were associated with actual lipid levels in the current study. As expected, (Table 4), the PRS were positively associated with the measured lipid levels in our cohorts. The results for all lipid PRS calculated at additional *p*-value thresholds and additionally adjusted for *APOE* ε4 or *APOC3* C homozygotes are available in the Supplementary (Tables S10, S11 and S12, respectively) The additional adjustments did not change the results significantly.

#### 3.3.3. Cardiovascular Health PRS and Exceptional Longevity 

**Figure 2:**
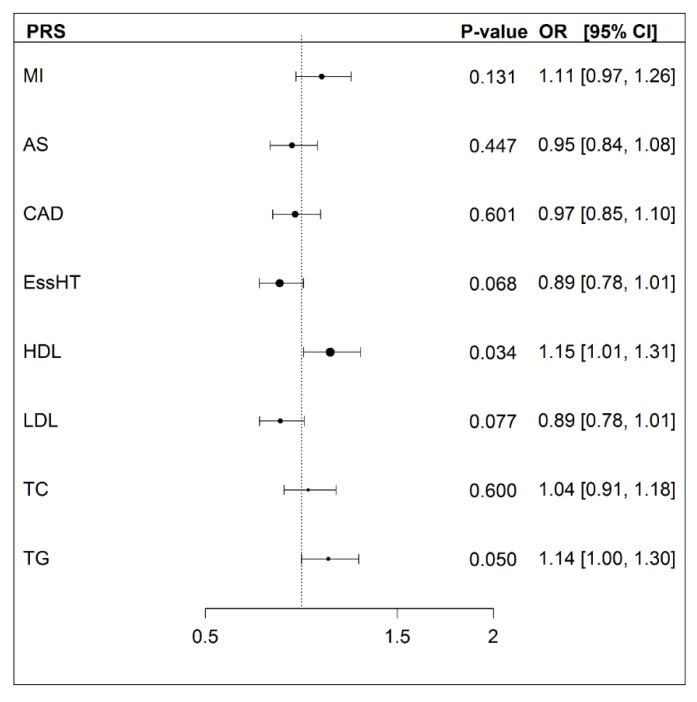
Forest plot showing the associations of different cardiovascular PRS with exceptional longevity. Notes. MI = myocardial infarction [31], AS = all stroke [32], CAD = coronary artery disease [33], EssHT = essential hypertension [www.nealelab.is], HDL = high-density lipoprotein, LDL = low-density lipoprotein, TC = total cholesterol, TG = total triglyceride [34]. Logistic regression analyses were adjusted for sex, comparing differences in PRS for ELL vs. controls. PRS were calculated using the relevant GWAS summary results with a *p*-value threshold < 5 × 10^−5^.

As shown in Figure 2, only two cardiovascular health PRS (HDL and TG) at the chosen *p*-value threshold of < 5 × 10^−5^ were nominally significant with exceptional longevity, with higher PRS associated with exceptional longevity. However, after Bonferroni correction for multiple testing, assuming eight independent tests, they no longer remained significant. The results for all cardiovascular health PRS calculated at additional *p*-value thresholds are available in the Supplementary (Table S13). Analyses were also adjusted for *APOE* ε4 and *APOC3* C homozygotes (Tables S14 and S15; when adjusting for *APOE* ε4, three cardiovascular health PRS (HDL, TG and myocardial infarction (MI)) were marginally significant but would not survive multiple testing correction.

##### PRS Genetic Overlap

**Figure 3:**
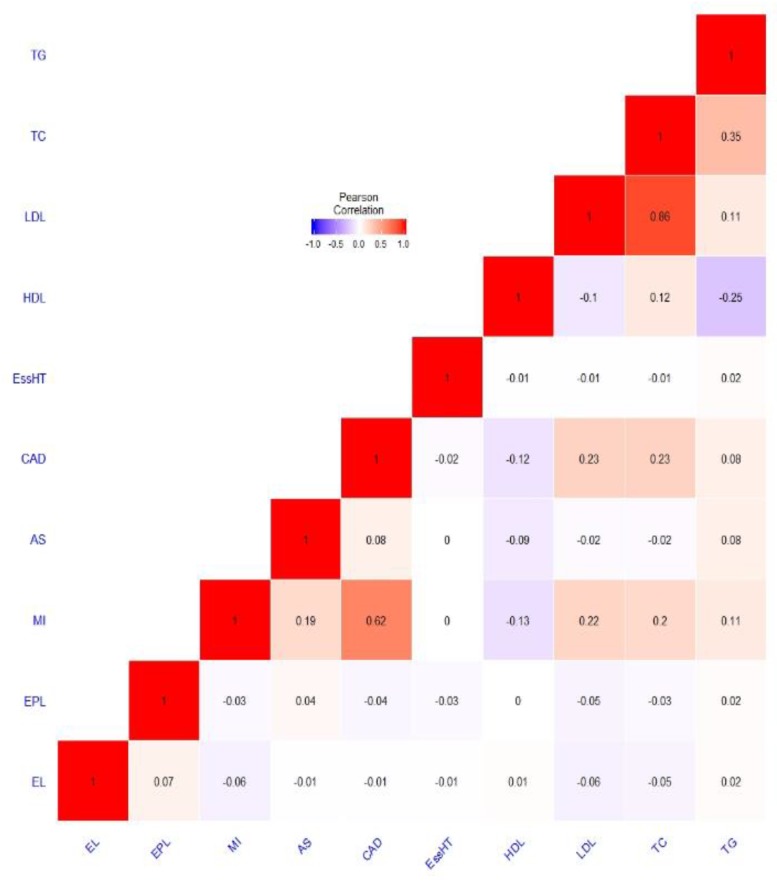
Pearson’s r correlation matrix comparing the genetic overlap between the investigated PRS. Note: EL = exceptional longevity [30], EPL = exceptional parental longevity [8], MI = myocardial infarction [31]; AS = all stroke, CAD = coronary artery disease [33], EssHT = essential hypertension [www.nealelab.is], HDL = high-density lipoprotein, LDL = low-density lipoprotein, TC = total cholesterol and TG = total triglyceride [34]. PRS were calculated using a *p*-value threshold of *p* < 5 × 10^−5^.

Figure 3 explores the genetic overlap between the investigated PRS. Exceptional parental longevity overlaps with the exceptional longevity PRS, although the correlation coefficient is small (r = 0.07, *p* = 0.009). *P*-values for the entire correlation matrix are provided in Table S16. Interestingly, LDL overlaps with both exceptional longevity (r = −0.06, *p* = 0.03) and exceptional parental longevity (r = −0.05, *p* = 0.04) in the expected direction. Myocardial infarction (MI) overlaps with exceptional longevity (r = −0.06, *p* = 0.03) but not exceptional parental longevity. Other significant correlations were observed between the cardiovascular risk factors and/or cardiovascular disease. For example, the MI PRS was correlated with the PRS for stroke, coronary artery disease, HDL, LDL, total cholesterol and triglycerides.

## 4. Discussion

In summary, this study did not confirm the hypothesis that ELL individuals have lower polygenic risk scores for cardiovascular-related phenotypes. Only the HDL cholesterol and triglyceride PRS were nominally significantly associated with ELL participants. In contrast and as expected, ELL individuals had higher polygenic risk scores for EL.

In regards to the associations of the various cardiovascular PRS with EL, no findings survived correction for multiple testing. This is despite validating the utility of the lipid PRS by confirming positive associations with measured lipid levels in our sample. Interestingly, the different lipid PRS were based on GWAS that found a large number of genome-wide significant loci (Table 1). ELL individuals had lower LDL and total cholesterol levels than controls in this study, but they did not differ on their respective PRS. This may suggest that environmental factors, perhaps lifestyle-related, influenced these lipid levels, which possibly promote longevity. The most significant finding in our study was for the HDL PRS, with higher scores associated with EL, which was in the expected direction (OR = 1.15, *p* = 0.034). Other findings that were nominally significant, or approached significance, that were also in the expected direction were the PRS for essential hypertension (OR = 0.89, *p* = 0.068) and LDL (OR = 0.89, *p* = 0.077). However, the direction of the relationship between the triglyceride PRS and EL was contrary to expectations (OR = 1.14, *p* = 0.050). Moreover, the relationships between EL and cardiovascular PRS constructed at less stringent GWAS *p*-value thresholds reached statistical significance with EL, although not always in the expected direction. In contrast, the UK Biobank study observed that extreme parental longevity (defined as at least one parent who survived to the top 1% of age at death) had lower polygenic risk for several cardiovascular health measures. Namely coronary artery disease, systolic blood pressure, body mass index, high-density lipoproteins, low-density lipoproteins and triglycerides. A similar result for HDL cholesterol and extreme parental longevity (EPL) by the UK Biobank to the current study was reported (OR = 1.08) [8]. Again, similar results were reported by the UK Biobank for LDL (OR = 0.89). However, the observed discrepancies between our analysis and the UK Biobank were most likely due to methodological differences, including the use of PRS that were based on different GWAS and *p*-value thresholds (*p* < 5 × 10^−5^ vs. *p* < 5 × 10^−8^, respectively). Additionally, sample sizes varied (e.g., cases: *n* = 294 vs. *n* = 1339, respectively) and there were differences in the definitions of EL (≥95 years vs. participants with at least one long-lived parent). 

This study confirmed significant associations between higher-longevity PRS and EL in our cohort using summary data from two different GWAS studies, Broer et al. [30] (≥90 years cases) and Pilling et al. [8] (cases had a mother who lived ≥98 and a father ≥95 years), with the results in the expected direction. Despite both of the longevity PRS not including any genome-wide significant variants (*p* < 5 × 10^−8^), PRS scores were significantly associated with EL in our sample. It should be noted that Pilling et al. [8], examining EPL, observed two genome-wide significant hits; however, they were not used in our PRS due to the quality control steps undertaken. Interestingly, 32 SNPs from the EL overlap with 123 SNPs in the EPL at the suggestive threshold of *p* < 5 × 10^−5^. EL PRS calculated at other GWAS *p*-value thresholds were all statistically significant with EL in our sample and showed strengthened associations (e.g., EL: OR increased from 1.2 to 1.8 at a threshold of *p* < 5 × 10^−5^ to <0.05). A significant but modest correlation between EL and EPL PRS suggests there is a slight overlap in genetic risk between these two longevity phenotypes. Moreover, when the whole genome (*p* ≤ 1) was considered for calculation of the EL and EPL PRS, the correlation coefficient increased to 0.46.

Limitations of this investigation include the relatively small sample size of the current study, which could result in low statistical power. This is demonstrated by a post hoc power analysis based on our observed sample sizes and parameters, which showed that an OR above 1.26 would have provided 80% power, assuming eight independent tests. However, some of the observed ORs were below 1.26 (GWAS *p*-value threshold < 5 × 10^−5^), which may have contributed to the observed non-significant results. This study did not examine sex differences, which may influence EL [9]. The other issue is the appropriate GWAS *p*-value threshold cut-off to use for the analyses. As can be seen from the results described, there is great variation in the results at different thresholds for the same PRS, with some findings in the expected direction, whilst others were contrary. Currently, there is no consensus regarding the use of thresholds, which can have a great influence on the interpretation of the results. 

## 5. Conclusions

Using the current GWAS data available, polygenic risk for cardiovascular-related phenotypes and disease calculated at the 5 × 10^−5^ threshold appear not to play a strong role in achieving EL. This is despite some evidence that cardiovascular pathways are involved, including lower prevalence of *APOE* ε4 carriers in ELL individuals [9,16]. On the other hand, at less stringent GWAS *p*-value thresholds, there were significant results observed but these were not always in the expected direction. Therefore, studies enrolling larger sample sizes are required to further explore the role of CVD-related genetic variants in EL. Sex and ethnic differences should also be examined. Other EL phenotypes could be investigated, including healthy EL and the use of more extremes of EL (e.g., supercentenarians), which may further reveal the extent of the contribution of cardiovascular genetic determinants to ageing successfully.

## Figures and Tables

**Table 1:** Sample characteristics of long-lived cases (≥95 years) and younger controls (55–65 years).

	Cases	Controls	*p*-Value
Cohort	SCS/Sydney MAS	HCS	
Sample size (*n*)	294	1105	N/A
Age range (mean ± SD)	95–106 (96.1 ± 4.1)	55–65 (60.3 ± 2.8)	N/A
*N* (%) males	90 (31)	518 (47)	N/A
*APOC3*^a^ C homozygotes, *n* (%)	120 (40.1)	475 (42.9)	0.503564
*APOE* ε4 carrier, *n* (%)	43 (14.6)	340 (30.8)	1.49 × 10^−8^
HDL (mean ± SD) (% missing)	1.47 ± 0.45 (17.1)	1.36 ± 0.37 (0.6)	0.148557
LDL (mean ± SD) (% missing)	2.69 ± 1.02 (17.3)	3.25 ± 0.91 (12.3)	1.23 × 10^−19^
TC (mean ± SD) (% missing)	4.75 ± 1.16 (17.0)	5.24 ± 1.03 (0)	6.37 × 10^−15^
TG (mean ± SD) (% missing)	1.31 ± 0.63 (17.0)	1.41 ± 1.12 (0.6)	0.492286

Notes: HDL = high-density lipoprotein, LDL = low-density lipoprotein, TC = total cholesterol, TG = total triglyceride. Raw mean values are reported. ^a^, *APOC3* SNP rs2542052.

**Table 2:** Longevity and cardiovascular-related risk and disease phenotypes examined, with the number of single nucleotide polymorphisms (SNPs) included in each polygenic risk score (PRS) at two GWAS *p*-value cut-offs.

Phenotype [GWAS ref]	N SNPs *p* < 5 × 10^−8^	N SNPs *p* < 5 × 10^−5^	Total Number of GWAS SNPs Available
Exceptional longevity			
Exceptional longevity [30]	0	32	184,562
Exceptional parental longevity [8]	0	123	476,093
Cardiovascular health			
Myocardial infarction [31]	35	107	19,607
Stroke [32]	11	226	471,632
Coronary artery disease [33]	96	447	460,589
Essential hypertension (http://www.nealelab.is)	2	107	476,069
HDL [34]	318	685	204,118
LDL [34]	301	652	202,316
Cholesterol [34]	367	797	204,123
Triglycerides [34]	238	592	201,879

Notes: SNP numbers are after QC steps for the PRS calculation, including removal of any SNPs with a poor imputation quality score (≤0.6) and linkage disequilibrium pruning. HDL = high-density lipoprotein, LDL = low-density lipoprotein.

**Table 3:** Associations between longevity EL and EPL PRS (*p*-value threshold < 5 × 10^−5^) and exceptionally long-lived cases versus controls.

	Odds Ratio (OR)	Standard Error [8]	*p*-Value
PRSEL [30]	1.20	0.068	0.00758
PRSEPL [8]	1.19	0.067	0.00804

Notes: EPL = exceptional parental longevity, defined as participants with a mother who lived ≥98 years and a father ≥95 years. EL = exceptional longevity, defined as participants who lived ≥90 years. Logistic regressions were adjusted for sex, comparing differences in PRS for ELL vs. controls. ORs expressed in PRS SD units.

**Table 4:** Associations of different PRS (*p*-value < 5 × 10^−5^) for lipid cardiovascular risk factors with measured lipid levels in the combined sample (55–106 years).

	Sample Size (*n*)	Beta (β)	Standard Error [8]	*p*-Value
PRSHDL	1331	0.261	0.024	3.94 × 10^−26^
PRSLDL	1196	0.200	0.028	3.81 × 10^−13^
PRSTC	1336	0.176	0.026	1.66 × 10^−11^
PRSTG	1335	0.254	0.026	2.63 × 10^−21^

Notes. HDL = high-density lipoprotein, LDL = low-density lipoprotein, TC = total cholesterol, TG = total triglyceride. Linear regression adjusted for age and sex, independent variable = PRS, dependent variable = lipid level. All lipid PRS were generated using summary results from Willer et al. [34].

## Supplementary Materials

The following are available online at https://www.mdpi.com/2073-4425/10/3/227/s1, Table S1: Description of GWAS data sets used for calculation of longevity PRS, Table S2: Description of GWAS data sets used for calculation of cardiovascular PRS, Table S3: The number of SNPs included in calculating a PRS at each *p*-value threshold; Table S4: Logistic regression results for longevity PRS and exceptional longevity in younger controls (55–65 years) vs. long-lived cases (>95 years) at different *p*-value thresholds, Table S5: Logistic regression results for longevity PRS and exceptional longevity in younger controls (55–65 years) vs. long-lived cases (>95 years) at different *p*-value thresholds without the *APOE* locus, Table S6: Logistic regression results for longevity PRS and exceptional longevity in younger controls (55–65 years) vs. long-lived cases (>95 years) at different *p*-value thresholds without the *FOXO3* locus, Table S7: Logistic regression results for longevity PRS and exceptional longevity in younger controls (55–65 years) vs. long-lived cases (>95 years) at different *p*-value thresholds without the *APOC3* locus, Table S8: Logistic regression results for longevity PRS and exceptional longevity in younger controls (55–65 years) vs. long-lived cases (>95 years) at different *p*-value thresholds adjusted for *APOE* ε4 carrier status, Table S9: Logistic regression results for longevity PRS and exceptional longevity in younger controls (55–65 years) vs. long-lived cases (>95 years) at different *p*-value thresholds adjusted for *APOC3*, Table S10: Associations for cardiovascular risk factors (lipids) with measured lipid levels in the combined sample (55–106 years) at various *p*-value thresholds, Table S11: Associations for cardiovascular risk factors (lipids) with measured lipid levels in the combined sample (55–106 years) at various *p*-value thresholds adjusting for *APOE*, Table S12: Associations for cardiovascular risk factors (lipids) with measured lipid levels in the combined sample (55–106 years) at various *p*-value thresholds adjusting for *APOC3*, Table S13: Logistic regression results for the association of cardiovascular PRS and exceptional longevity PRS in younger controls (55–65 years) vs. long-lived cases (>95 years) at different *p*-value thresholds, Table S14: Logistic regression results for the association of cardiovascular PRS and exceptional longevity PRS in younger controls (55–65 years) vs. long-lived cases (>95 years) at different *p*-value thresholds adjusting for *APOE*, Table S15: Logistic regression results for the association of cardiovascular PRS and exceptional longevity PRS in younger controls (55–65 years) vs. long-lived cases (>95 years) at different *p*-value thresholds adjusting for *APOC3* and Table S16: *p*-values for Pearson’s correlation matrix comparing the genetic overlap between the investigated PRS (*p*-value threshold of 5 × 10^−5^).
